# Brief parental self-efficacy scales for promoting healthy eating and physical activity in children: a validation study

**DOI:** 10.1186/s12889-021-10581-7

**Published:** 2021-03-19

**Authors:** Åsa Norman, Julie Wright, Emma Patterson

**Affiliations:** 1 Department of Global Public Health, Karolinska Institutet, Tomtebodavägen 18 A, SE-171 77 Stockholm, Sweden; 2 Department of Exercise and Health Sciences, University of Massachusetts, Boston, MA USA; 3 Region Stockholm, Centre for Epidemiology and Community Medicine, SE-171 77 Stockholm, Sweden

**Keywords:** Invariance, Psychometric evaluation, Confirmatory factor analysis, Construct validity, Parental support, Sweden, Schoolchildren

## Abstract

**Background:**

Brief scales to measure parental self-efficacy (PSE) in relation to children’s obesogenic behaviours have not been developed and validated using more rigorous methodology such as invariance testing, limiting their generalisability to sub-groups.

This study aimed to assess the construct validity and measurement invariance of brief PSE scales for children’s intake of vegetables, soft drinks, and sweets, and physical activity.

**Methods:**

Parents (*n* = 242) of five-to-seven-year-old children in disadvantaged and culturally diverse settings in Sweden responded to a questionnaire in Swedish with 12 items assessing PSE in relation to healthy and unhealthy behaviours. Construct validity was assessed with confirmatory factor analysis, invariance testing compared the scales by groups of parental sex, education, and child weight status. Criterion validity was evaluated using objective measures of children’s physical activity and semi-objective measures of diet.

**Results:**

Two-factor models showed moderate to excellent fit to the data. Invariance was supported across all groups for healthy behaviour scales. Unhealthy behaviour scales were invariant for all groups except parental education where partial metric invariance was supported. Scales were significantly correlated with physical activity and diet.

**Conclusion:**

This study provides preliminary evidence for the validity of brief PSE scales and invariance across groups suggesting their utility for research and clinical management of weight-related behaviours.

**Supplementary Information:**

The online version contains supplementary material available at 10.1186/s12889-021-10581-7.

## Introduction

Child obesity has increased globally during the last decades and is one of the most serious public health concerns [1–3]. In high-income countries such as Sweden, data suggests that a plateau in child obesity rates has been reached: levelling off on high levels in the overall population, but with a continued increase seen in children in socioeconomically deprived settings [4–6]. Treatment and prevention early in life is key as obesity has been seen to track from childhood to adolescence and adulthood [7, 8]. Healthy dietary intake and physical activity behaviours are important modifiable lifestyle factors for managing and preventing child obesity, and the literature states that interventions should focus on behaviour change or promotion of such healthy habits [9, 10]. Obesity management and prevention interventions for younger children are highly reliant on caregivers of the child, who function as gatekeepers for healthy behaviours in the home environment, i.e. situations where parents and children spend time together. It is therefore important to develop interventions and programs that can support parents’ efforts to promote children’s healthy behaviours and reduce unhealthy behaviours in the home environment [10–12]. Such interventions could target specific mechanisms in parenting behaviour for children’s healthy behaviours as described in underlying theories for behaviour formation and change.

Social Cognitive Theory is widely used to explain behaviour and guide development of interventions targeting obesity-related behaviours in children [13]. Self-efficacy is a central construct in the formation of behaviours according to this theory [14, 15] and has been identified as an important mechanism for change in management and prevention interventions targeting child obesity [16, 17]. Albert Bandura, credited with developing Social Cognitive Theory, has defined self-efficacy as “beliefs in one’s capabilities to organize and execute the courses of action required to produce given attainments” ([14], p. 3). As parents play such an important role in their children’s behaviour, a type of self-efficacy, parental self-efficacy (PSE), has been defined as parents’ beliefs regarding their own parenting capabilities to support their children in developing healthy behaviours throughout the course of childhood [14]. PSE has been associated with children’s dietary and physical activity (PA) behaviours in a number of previous studies, which indicates its potential importance in prevention of child obesity [18–22]. Bandura has published specific guidelines to be considered in the construction of scales to measure self-efficacy [23], and several scales have been developed to capture the PSE construct in relation to children’s dietary intake behaviours [19–21, 24–30] and PA behaviours [20, 21, 25–31] in a valid and reliable manner. Certain scales have also been developed for specific sub-groups of parents such as for parents of children with overweight children [29] or obesity [28] and specific ethnic groups [31] and low socioeconomic status [24, 30]. The guidelines state that scales should include items with a graduation of challenge for the respondent, reflecting situations in which the respondents may find it difficult to perform a behaviour [23]. However, few of the currently evaluated scales include contextually challenging situations [26–28, 30]. In addition, few scales measuring PSE in relation to children’s PA behaviours have used objective measurements of children’s PA to assess criterion validity [20, 30, 31].

Bandura suggested that a number of items per behaviour domain need to be included in order to cover different aspects of the behaviour domain [23]. While there is no ideal number of items, no fewer than three items can adequately represent a latent variable (construct) while adding more items can lead to response bias due to respondent fatigue. As a general rule, scales should be brief to prevent participant burden in research, yet most scales evaluated to date include a large number of items. Scales for use in the clinical setting must also be brief. Clinicians, whether in primary care, child- or school health care, can play a key role in supporting families to manage or prevent child obesity, and several official guidelines now recommend that clinicians should focus on providing strategies to families to support family-based behaviour change [32, 33]. Valid tools to be able to assess different aspects of parental motivation, such as PSE would be useful. However, primary care clinicians in particular often have very limited time for each patient meeting, something they find frustrating and challenging, and which may impact on quality of care [34–36]. Thus, there is a need for brief scales to facilitate primary care clinicians in their time-constrained patient meetings.

Furthermore, an important aspect of the usefulness of scales in research is that the underlying concept captured by the scale means the same thing across groups, i.e. that the scale functions equally across specified groups differing in an important characteristic. This can be statistically tested through measurement invariance testing, which is an important step in assessing scale validity [37, 38]. Groups of importance on which to test a PSE scale can be, for example, mothers and fathers, parents with higher or lower educational levels, or parents of children with normal weight or obesity. Unless measurement invariance has been established, scales should be used with caution in different groups or to compare groups [37, 38]. To our knowledge, no PSE scale for children’s obesogenic behaviours has yet been tested for measurement invariance across different parental groups.

In summary, there is a lack of validated scales measuring parental self-efficacy regarding children’s dietary intake or physical activity behaviours that are brief enough for use in research and clinical practice, that include contextually challenging situations for parents, and which have been tested for measurement invariance. This study aimed to evaluate the validity, internal consistency, and measurement invariance of brief scales to measure parental self-efficacy for children’s physical activity, and for intake of healthy and unhealthy food in the home environment. Specifically, we hypothesised that two factor models of PSE for promoting children’s healthy behaviours and limiting children’s unhealthy behaviours would fit data. Furthermore, we hypothesized that measurement invariance would be supported across groups differing in sex, parental educational level, and child weight status. We also hypothesised that PSE would be positively correlated with healthy behaviours, and negatively correlated with unhealthy behaviours.

## Methods

### Setting and participants

Participants in this study comprised parents participating in the baseline measurement of the Healthy School Start Plus programme (HSSP) [39]. The HSSP is a parental support programme to promote healthy dietary and physical activity behaviours and prevent overweight and obesity in children in disadvantaged areas in Sweden [39], focusing specifically on behaviours in the home environment. The HSSP is designed to be run during the first year of school, when children are 5–7 years old. It is based on Social Cognitive Theory [15] and is described in more detail in a published study protocol [39]. Although in the present study we were not aiming to validate the scales for use by disadvantaged families only, we used the HSSP sample for pragmatic reasons and also to ensure that there was a good spread of education levels among participants in the present study. This is otherwise difficult to achieve as participants with disadvantaged characteristics, such as lower level of education, are difficult to recruit and retain in research [40, 41].

The HSSP was a cluster-randomised trial in 17 schools in seven different municipalities during 2017–2019. Recruitment was conducted in the following manner: A convenience sample of municipalities in mid-Sweden were recruited by contacting key persons (head school nurses, educational boards, and public health practitioners). Interested municipalities provided contact details for primary schools. Schools where less than 50% of parents had a university education (which is below the national average) were eligible for inclusion and invited to participate as the intervention targeted disadvantaged families. All parents in the 17 schools which consented to be involved were then invited to participate. Information was provided in writing and orally, through information meetings, face-to-face at schools, and via telephone. A total of 352 families in the 17 schools consented to participate in the main HSSP trial. The parents included in this present study comprised all mothers and fathers (*n* = 242) who filled in the PSE questionnaires during the baseline measurements of the HSSP trial in September–October 2017. If both parents responded to the questionnaire individually, both mothers and fathers of the same child were included. All parents provided written consent and the study obtained ethical approval (No. 2017/711–31/1) from the Regional Ethical Review Board in Stockholm.

### Procedures

This validation study included multiple phases. First, a pool of items was generated from existing measurement tools, with special consideration given to the scales developed by Wright and colleagues [26]. Second, cognitive testing was performed to identify items suitable for a Swedish, disadvantaged setting. Then, the resulting twelve items comprised the baseline questionnaire that was administered online via the HSSP trial. Finally, these answers were used to evaluate the validity of the scale, by examining internal consistency, construct and criterion validity, and finally measurement invariance.

### Development of the specific PSE scale

When developing scales to measure self-efficacy, Bandura [23] recommends that items should be specific to the behaviour domain in focus, and that several items should be included in order to capture different aspects of the domain. In addition, items should include situations that present the respondent with contextual challenges, and have a response scale that ranges from 0 to 10 [23]. The development of the PSE questionnaire used in this study is primarily based on the questionnaire developed and tested by Wright et al. [26]. The Wright questionnaire was developed for a US context and comprises four separate behaviours: 1) physical activity 2) fruits and vegetables 3) sugary drinks, and 4) fruit juice, with four items for each behaviour [26].

The items in the present study differ from the Wright scale in five major ways. 1) Fruit juice was omitted, and sweets were included instead, as sweets are an important source of low-nutrient density energy in the diets of children in Sweden [42]. We felt there was overlap between fruit juice and sugary drinks and that sweets, which are another distinct food group that parents often wish to limit, were more relevant to include. 2) Fruit was also omitted (i.e. only vegetables were included) as studies have shown that the support needed to get children to eat more vegetables differs from that needed for fruit [43, 44]. 3) The number of items per behaviour was reduced in order to minimise participant burden as well as to improve parsimony. 4) An 11-point response scale was used instead of a 6-point scale. The 11-point response scale was chosen in order to capture a greater variation in response scores, as suggested by Bandura [23]. 5) Items were adapted to the Swedish context, where parents may be challenged by different situations compared to the US. For example, in Sweden, all children are provided with school food at no cost, and it is generally of very good quality, with little energy-dense, nutrient-poor food available during the school day. Furthermore, the notion of “Saturday sweets” is widespread, i.e. that sweets should ideally only be consumed 1 day per week. Although whether this type of limit (if enforced) has a positive influence, leading to lower intake of sweets, or negative, whereby restriction creates increased interest and desire, is unclear. In addition, many physical activity options are available for free or at low cost to children and families. For example, free outdoor playgrounds are readily available, many organised sports are volunteer-run and part-subsidised, and Sweden practices the Right of Public Access, which makes it possible for everyone to spend time outdoors in parks, countryside or woods. Thus, the four behaviour domains that we wanted to capture were the following: 1) supporting the child’s PA for 1 hour per day on weekends, 2) influencing the child’s daily vegetable intake, 3) limiting the child’s weekly intake of soft drinks, and 4) limiting the child’s weekly intake of sweets/chocolate (see Table 1 and Supplementary file 1). Behaviour domains were focused on child behaviour in the “home environment”, i.e. time spent outside of school or after school care, but not restricted to time spent in the physical home. Thus, what is meant is mornings, afternoons, evenings, and weekends where the parents potentially spend time with their child and have the possibility to influence the child’s behaviours.

**Table 1:** Description of factors, items, item means, factor loadings, and internal consistency in the four behaviour domains tested

Behaviour/item Basic stem: *“How certain are you that you can …*”	Mean (SD)	Standardised factor loading	Cronbach’s Alpha
Healthy behaviours			
Physical activity			0.92
“*…*. *make sure that your child is physically active in such a way that he/she gets a little sweaty or out of breath for at least 1 h during the day?”*			
1. … when there are many other things to do	5.58 (2.76)	0.92	
2. … when you are tired	5.43 (2.71)	0.93	
3. … when the weather is bad	5.17 (2.75)	0.84	
Vegetables			0.92
*“… influence your child to eat at least 2 servings of vegetables at home each day?”*			
4. … when you are too tired to prepare them	5.59 (3.27)	0.91	
5. … when other family members don’t want to eat vegetables	5.86 (3.13)	0.92	
6. … when you are eating out at a restaurant	5.05 (2.98)	0.82	
Unhealthy behaviours			
Soft drinks			0.84
*“… limit how much soft drinks and sap your child drinks so that your child does not drink more than 2 glasses (3 decilitres) per week”*			
7. … when other family members drink it	6.75 (3.49)	0.72	
8. … when you eat at a restaurant	6.96 (3.20	0.82	
9. … when your child wants it	7.82 (2.82)	0.85	
Sweets			0.81
*“… limit how much sweets/chocolate your child eats so that your child does not eat more than 100 g/1.5 decilitre sweets per week”*			
10. … when other family members eat it	6.80 (3.29)	0.63	
11. … when your child refuses to eat food	8.59 (2.50)	0.89	
12. … when your child wants it	8.26 (2.49)	0.93	

Item development was undertaken as follows. First a pool of items was developed by the researchers, based on the original items from the Wright scales, previous studies in the Swedish context regarding parents’ perceptions on difficult situations related to children’s dietary intake and physical activity behaviours in the home environment [45, 46], experiences from previous assessments of PSE scales in the Swedish context [20, 30], and clinical experiences from work with families to treat or prevent child obesity in the Swedish context. The initial item pool consisted of seven to nine items per behaviour domain, which were then reviewed by six experts in parenting, diet and physical activity research and further narrowed to a pool of five items per behaviour domain to be tested.

Cognitive testing was used to assess the suitability of items in the target group [47]. Testing was completed with eleven parents. Eight were mothers, seven were born outside of Sweden, and eight had ≤12 years of schooling. Testing was conducted individually by telephone. The parent was instructed to think aloud whilst reading and answering the questionnaire [47]. A research assistant used probing to elicit comprehensibility, relevance, and how the item challenges related to the parent’s everyday life. Parents voiced comments and suggested revisions of items to improve comprehension. The comments to each of the five items were presented to three of the previously consulted experts in parenting, dietary intake and physical activity research and discussed between the main author, from which a final decision on items to be included in the scales was taken. Testing resulted in a final version of the scale with three items per behaviour, identified as the most contextually relevant, and comprising three situations of different levels of challenge (see Table 1 for stems and items).

For the HSSP trial, a questionnaire was created with these items. All behaviours were illustrated with either a picture indicating the specified amount of the food or written examples of the activity. Items were not presented in order of ascending difficulty but were mixed. All questionnaires in the HSSP trial were web-based and accessed via the project website (www.enfriskskolstartplus.se) and responded to in Swedish.

### Measurements

#### Dietary intake

Children’s dietary intake was measured using a mobile phone photography-based method developed for use in the HSSP trial [48]. Parents took photos of all food and drink consumed and left-over for 3 days, including one weekend day, and sent them to the researchers via multimedia messaging service (MMS). The volume of selected foods, including fruits, vegetables, sweets, and soft drinks, present in the photos was coded by a nutritionist trained in the method. In a relative validation study [48], in yet another group of parents with a variation in educational and immigrant backgrounds (*n* = 19), the photo method was compared to 24-h recalls (conducted by licenced dietitians) on 3 days during a week. The photo method showed acceptable validity relative to the reference method of 24-h recalls, with correlations between the volumes of these four food groups as assessed by the photo method and by the reference method ranging from 0.562 to 0.688. Under-reporting occurred but was non-differential.

#### Physical activity

Children’s physical activity was measured using accelerometry (GT3 X+, Actigraph, LCC, Pensacola, USA) which is considered a valid, reliable, and objective method [49]. Children wore hip-worn accelerometers during wake time for 7 days. ActiLife Data Analysis, version 6.5.2 was used to analyse data. PA was assessed between 7 am and 9 pm with the epoch length set to 5 s. Children who registered ≥500 min of activity per day were included. Non-wear time was defined as 60 min of consecutive zeros, allowing for 2 min of non-zero interruptions. Moderate to vigorous intensity (MVPA) was defined as activity > 2296 counts per minute (CPM) [50]. Time (mins) spent in MVPA was calculated for weekend days, and for weekdays outside of school, i.e. excluding 8 am to 4 pm.

#### Weight status

Body composition was measured by trained research assistants using SECA instruments to a level of precision of 1 mm for height, and 100 g for weight. Weight status was classified according to the International Obesity Task Force definitions [51].

#### Data analysis

Descriptive statistics were used to explore item-specific normality, and participant characteristics are presented as means, standard deviations (SD) and percentage (%).

Confirmatory factor analysis (CFA) was used to assess construct validity. CFA is used to test a hypothesised model against previous theoretical and empirical reports in the literature [52]. As self-efficacy is a well-established theoretical construct [14, 23], numerous psychometric tests of self-efficacy scales have been undertaken [19–21, 24, 26–31]. The current scale is based on a previously tested scale using CFA [26], and as the current scale also has a clearly hypothesised structure, CFA was considered to be the most appropriate assessment method. To test model fit, the six healthy behaviour items (for PA and vegetable intake) were tested in one model and the six unhealthy behaviour items (for soft drinks and sweet intake) in another. The healthy and unhealthy behaviour domains were grouped based on theoretical proximity of the behaviours. For example, associations between high vegetable intake and high PA have been found [53, 54], and also clustering of unhealthy dietary intake behaviours [55]. Maximum Likelihood estimation was used in the CFA. Model fit was evaluated with four fit indices: chi-squares/degrees of freedom ratio, comparative fit index (CFI), root mean squared error of approximation (RMSEA) with 90% confidence interval, and standardised root mean residuals (SRMR). The chi-square/degrees of freedom ratio is sensitive to sample size, but in general, a good model fit is indicated by a non-significant *p*-value (> 0.05). For CFI, a value of ≥0.9 indicates acceptable model fit and ≥ 0.95 good fit. For RMSEA, < 0.05 indicates good model fit, < 0.08 acceptable fit, and 0.08 to 0.10 mediocre fit. For SRMR, < 0.1 is considered acceptable model fit. For data deviating from the multivariate normality assumption with a value > 5.00 [56], Maximum Likelihood estimations with bootstrapping using 1000 samples was used to obtain an accurate estimation of standard errors. Bias-corrected confidence interval was set to 95%. In addition, the Bollen–Stine bootstrap *p*-value was used to indicate model fit, where *p* > 0.05 indicates good model fit [57]. Internal consistency was assessed using Cronbach’s alpha, where an alpha > 0.7 is considered acceptable, > 0.8 considered good, and > 0.9 considered excellent. Internal consistency was assessed for the two-factor models (healthy and unhealthy behaviours), and for the four separate factors (PA, vegetable, soft drinks, and sweets).

Invariance testing was conducted to assess whether the scales could be considered to function similarly across groups: between mothers and fathers, between parental educational ≤ and > 12 years of schooling, and between children with overweight/obesity and normal/underweight. Ideally, to establish invariance, three levels of invariance - configural, metric, and scalar - should be fulfilled. Configural invariance implies that the model, including the manifest items and the latent construct, holds across groups. Metric invariance implies equivalence of item loadings across groups, and scalar invariance implies equivalence of item intercepts across groups [37]. Invariance testing was conducted in accordance with the multigroup procedure in AMOS described by Byrne [58], using the parameterization approach of constraining one item to 1 and the corresponding intercept to 0.

Metric invariance was tested by constraining item loadings to be equal across groups, and scalar invariance was tested by further constraining item intercepts to be equal across groups. Configural invariance was assessed by evaluating overall model fit. The remaining two tests for invariance, metric and scalar, were nested in the multi-group procedure, and thus, each model was compared to the previous one regarding model fit. If model fit was not significantly worse, this was considered evidence of invariance across groups. To compare models, the delta chi-square ratio, delta CFI, delta SRMR, and delta RMSEA were used. Acceptable model fit was set to insignificant delta chi-square ratio (*p* > 0.05), ≤ − 0.01 delta CFI, ≤0.015 delta RMSEA, and ≤ 0.03 delta SRMR [37]. If invariance was not supported, a test for partial invariance was conducted by releasing constraints for one item at a time in a backwards approach [37].

Criterion validity was explored by Pearson correlations, and assessed for the two-factor models (healthy and unhealthy behaviours), and for the four separate factors (PA, vegetable, soft drinks, and sweets). Regarding the two-factor models, scores for PSE for healthy behaviours were correlated with objectively measured child behaviour regarding both mean minutes in MVPA, and intakes in decilitres (dl) of vegetables, and scores for PSE for unhealthy behaviours were correlated with both intakes (dl) of soft drinks and sweets. The separate factors were assessed in the same manner where PSE for child PA was correlated with objectively measured child MVPA, and PSE for child intake of vegetables, soda, and sweets were correlated with child intake of the corresponding food group measured through the photo method.

All data analyses were performed using SPSS version 25.0 (IBM Corp., Armonk, NY, USA) and AMOS version 25.0 (IBM Corp., Armonk, NY, USA) was used specifically for the confirmatory factor analysis (CFA). The level of statistical significance was set to *p* < 0.05.

## Results

In total, 242 parents (141 mothers, 101 fathers) of 155 individual children responded to the PSE scale. Of the parents, 40% had 12 years or less of schooling, 29% were born outside of Sweden, and the overall majority were married or co-habiting and were employed. Children were on average 6.2 (SD 0.3) years old; 47% were boys and 25% were overweight or obese (Table 2).

**Table 2:** Characteristics of parents and children participating in the study

	Mean (SD)/%
Parents *n* = 242	
Mothers	58%
Education	
≤9 years	7%
> 9 years ≤12 years	33%
Technical	19%
University	41%
Born in Sweden	71%
Married/cohabiting	91%
Employed	91%
Children *n* = 155	
Girls	53%
Age	6.2 (0.3)
Underweight^a^	6%
Normal weight^a^	69%
Overweight^a^	14%
Obese^a^	11%

^a^: according to Cole et al. 2012 [51]

### Confirmatory factor analysis

**Fig. 1:**
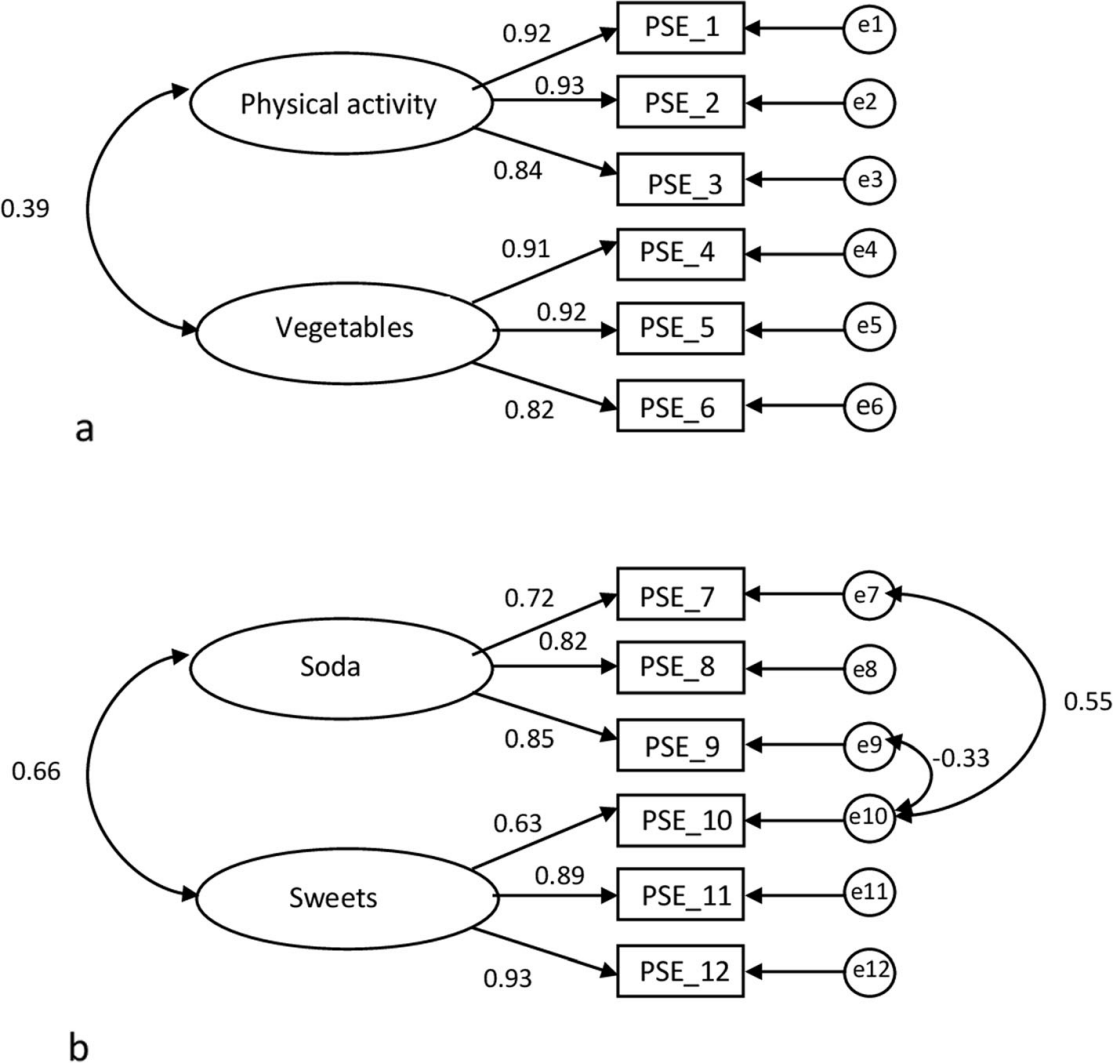
**a** and **b** Models assessed for construct validity, **a** model for healthy behaviours (physical activity and vegetables), and **b** model for unhealthy behaviours (soft drinks and sweets). Figures include standardised estimates for items, correlation between latent factors, and correlation of error terms (Fig. 1b)

In the CFA, two-factor models were tested: healthy behaviours (PA and vegetables) and unhealthy behaviours (soft drinks and sweets), according to Fig. 1a and b. Standardized factor loadings ranged between 0.63 and 0.94, with the majority > 0.8 (Fig. 1a, b, and Table 1). While the healthy and unhealthy models violated the multivariate assumption with values > 5.00, where this violation may impact on model fit indices and factor loadings, the Bollen-Stine bootstrap yielded insignificant Bollen-Stine *p*-values (Table 3), meaning that the models showed approximate fit to data. For the healthy behaviour model, the CFA yielded excellent model fit with an insignificant chi-square ratio value (*p* = 0.74), SRMR = 0.01, CFI = 1.00, and RMSEA = 0.00 (0.00;0.06). As the unhealthy behaviour model yielded a poor model fit at first, two error terms were allowed to correlate (Fig. 1b). This resulted in moderate model fit for the unhealthy behaviour model with a significant chi-square ratio value (*p* = 0.004), SRMR = 0.04, CFI = 0.99, and RMSEA = 0.09 (0.05; 0.14) (Table 3). In the two-factor models, the correlation between the different sub-factors, vegetables and PA was 0.39 in the healthy behaviour model, and between soft drinks and sweets was 0.66 in the unhealthy behaviour model.

**Table 3:** Results from the confirmatory factor analysis of the two-factor models: healthy and unhealthy behaviours

Model	χ2	df	p	SRMR	CFI	RMSEA	lower	upper	Bollen-Stine (p)	Cronbach’s Alpha	Factor correlation
Healthy behaviours (PA & vegetables)	5.158	8	0.741	0.0127	1	0	0	0.055	0.856	0.858	0.39
Unhealthy behaviours (soft drinks & sweets)	18.876	6	0.004	0.044	0.985	0.094	0.048	0.144	0.084	0.862	0.66

*PA* physical activity, *χ2* Chi-Square, *df* degrees of freedom, *SRMR* Standardized Root Mean Residual, *CFI* Comparative fit index; *RMSEA* Root mean squared error of approximation, *Bollen-Stine (p)* Bollen–Stine bootstrap p-value indicating model fit

### Internal consistency

Cronbach’s alpha for both the healthy and the unhealthy behaviours was 0.86 (Table 3). Cronbach’s alpha for the four behaviours separately were: PA 0.92, vegetables 0.92, soft drinks 0.84, and sweets 0.81 (Table 1).

### Scale invariance

Measurement invariance (configural, metric and scalar) was tested in groups differing by parental sex, level of education, and child weight status, as shown in Table 4. Evidence for configural invariance was found for all three groups regarding both healthy and unhealthy behaviours. Metric invariance was supported for parental sex and child weight status for both unhealthy and unhealthy behaviours. A significant delta chi-square ratio (*p* < 0.05) was found for unhealthy behaviours regarding child weight status, but the remaining fit indices were good and therefore the model was accepted. Regarding parental education, metric invariance was not supported for unhealthy behaviours, with a significant delta chi-square ratio (*p* < 0.01) and delta CFI > -0.01. A partial invariance test, releasing equality constraints one item at a time, provided support for partial invariance when all items were constrained to be equal except for item 9. Evidence of scalar invariance was found for all three groups regarding both healthy and unhealthy behaviours. A significant delta chi-square ratio (*p* < 0.05) was found for unhealthy behaviours regarding parental education, but the remaining fit indices were good and therefore the model was accepted.

**Table 4:** Results of invariance testing for the two-factor models: healthy and unhealthy behaviours

Model	χ2 (df)	CFI	RMSEA (90% CI)	SRMR	Model comparison	Δχ2 (Δdf)	ΔCFI	ΔRMSEA	ΔSRMR	Decision
**Parent sex**										
Mother *n* = 141, father *n* = 101)										
Healthy behaviours										
M1 Configural	14.233 (16)	1	0 (0; 0.53)	0.0236						Accept
M2 Metric	17.676 (20)	1	0 (0; 0.48)	0.0238	M1	3.443 (4)	0	0	0.0002	Accept
M3 Scalar	25.399 (26)	1	0 (0; 0.50)	0.0239	M2	7.723 (6)	0	0	0.0001	Accept
Unhealthy behaviours										
M4 Configural	20.244 (12)	0,991	0.054 (0.000; 0.093)	0.0517						Accept
M5 Metric	27.163 (16)*	0,987	0.054 (0.012; 0.088)	0.0511	M4	6.919 (4)	−0.004	0.0	−0.0006	Accept
M6 Scalar	39.736 (22)*	0,98	0.058 (0.027; 0.086)	0.0507	M5	12.573 (6)	−0.007	0.004	− 0.0004	Accept
**Parent education**										
(High *n* = 141, low *n* = 96)										
Healthy behaviours										
M7 Configural	29.531 (16)*	0.988	0.060 (0.023; 0.093)	0.0227						Accept
M8 Metric	31.089 (20)	0.990	0.049 (0; 0.08)	0.0232	M7	1.558 (4)	0.002	−0.011	0.0005	Accept
M9 Scalar	36.909 (26)	0.991	0.042 (0; 0.071)	0.0233	M8	5.82 (6)	0.001	−0.007	0.0001	Accept
Unhealthy behaviours										
M10 Configural	23.173 (12)*	0.987	0.063 (0.021; 0.101)	0.0544						Accept
M11 Metric	38.827 (16)*	0.974	0.078 (0.047; 0.109)	0.051	M10	15.654 (4)*	−0.013	0.014	−0.0033	Reject
M112 Partial metric	27.108 (15)	0.986	0.059 (0.019; 0.093)	0.0537	M10	3.935 (3)	−0.001	− 0.004	− 0.0007	Accept
M12 Scalar	42.264 (22)*	0.977	0.063 (0.033; 0.091)	0.0511	M11:2	15.156 (7)*	−0.009	0.04	− 0.0026	Accept
**Child weight status**										
(Under/normal *n* = 177, OWOB *n* = 60)^a^										
Healthy behaviours										
M13 Configural	17.991 (16)	0.998	0.023 (0; 0.067)	0.0211						Accept
M14 Metric	22.941 (20)	0.997	0.025 (0; 0.064)	0.0216	M13	4.95 (4)	−0.001	0.002	0.0005	Accept
M15 Scalar	28.665 (26)	0.998	0.021 (0; 0.057)	0.0215	M14	5.724 (6)	0.001	−0.003	− 0.0001	Accept
Unhealthy behaviours										
M16 Configural	29.437 (12)*	0.981	0.079 (0.043; 0.115)	0.0437						Accept
M17 Metric	40.460 (16)*	0.973	0.081 (0.050; 0.112)	0.0448	M16	11.023 (4)*	−0.008	0.002	0.0011	Accept
M18 Scalar	45.171 (22)*	0.975	0.067 (0.039; 0.095)	0.0448	M17	4.711 (6)	0.002	−0.014	0	Accept

*p < 0.05, χ2 = Chi-Square, *CFI* Comparative fit index, *RMSEA* Root mean squared error of approximation, *SRMR* Standardized Root Mean Residual, Model comparison: the model which the current model is compared with to interpret acceptance or rejection of invariance, Δ: delta, Decision: accepting or rejecting the hypothesis that the model is invariant, High education: > 12 years of schooling, Low education: ≤12 years of schooling, ^a^: weight status according to Cole et al. 2012 [51], underweight, normal weight overweight (OW), obesity (OB)

### Criterion validity

Correlations between factors and child behaviours are shown in Table 5. Significant correlations in the hypothesised direction (i.e. greater scores for PSE associated with healthier behaviour) were found between mean scores of relevant two-factor models and separate factors and child behaviour regarding physical activity outside of school time on weekdays, intake of vegetables, and soft drinks. Correlations were non-significant, but in the hypothesised direction, between the healthy behaviour two-factor model and the separate PA factor and physical activity on weekends, and intake of sweets for the separate sweets factor. One exception in the criterion validity assessment was the correlation between the unhealthy behaviour two-factor model and the intake of sweets where the correlation was in the non-hypothesised direction, though very low (0.008).

**Table 5:** Correlations between factor mean scores of two- and four-factor models and children’s objectively measured physical activity, and dietary behaviours

Factor mean score	Child behaviour				
	MVPA average mins/day weekend	MVPA average min/day, weekday outside of 8 am-4 pm	Vegetable intake dl/day	Soft drinks intake dl/day	Sweets intake dl/day
n	194	217	237	237	237
Two-factor models					
Healthy behaviours	0.153*	0.175**	0.123	–	–
Unhealthy behaviours	–	–	–	− 0.257**	0.008
Four-factor models					
Physical activity	0.130	0.189^**^	–	–	–
Vegetables	–	–	0.242^**^	–	–
Soft drinks	–	–	–	−0.222^**^	–
Sweets	–	–	–	–	−0.039

* *p* < 0.05

** *p* < 0.01

- Not tested

*MVPA* moderate to vigorous physical activity

*dl* decilitre

Results of Pearson correlation

## Discussion

The psychometric assessment of the PSE scales in relation to children’s obesity related behaviours provides preliminary evidence for the validity of these scales. Our results suggest that the scales had moderate to excellent construct validity and internal consistency, and, for the most part, behaved as expected when compared to objectively measured dietary and physical activity behaviours. Also, the scales proved to be invariant across groups of parents that differed by sex, by child weight status, and was partially invariant across parental educational level. These PSE scales may be useful for intervention researchers that investigate effects on PSE as an outcome or a mediator, to compare group means of PSE, or for clinicians who meet families that need support with weight-related behaviours.

There are currently a number of PSE scales that have been tested for validity with different degrees of rigour in the literature [19–21, 24, 26–31]. Of the currently evaluated scales, none have tested measurement invariance, only three have tested criterion validity using objectively measured PA [20, 30, 31], and few scales include contextually challenging situations [26–28, 30]. In this study, there is evidence for the scales’ criterion validity given that the majority of the correlations were in the hypothesized direction and significant. The magnitude of the correlation coefficients were similar to scales that assessed criterion validity using levels of the target behaviour as the criterion [20, 26, 27, 30].

Regarding the scales that have included challenging situations, there are obvious differences between those scales and the scales assessed in this study. In comparison with the scales developed by Wright et al. [26], from which the scales of this study were derived, it is important to keep in mind that a number of changes were made to the current scale: it includes PSE for limiting sweets which is not included in the Wright scales, it has a smaller number of items (three items per behaviour), it has a wider response scale to capture a greater range of PSE. An overall comparison between the current scales and the Wright scales show that they performed similarly in the psychometric testing with good model fit in the CFA. The current scales show somewhat higher factor loadings and internal consistency, but with a greater range. Bohman et al. [27] developed another scale for measuring PSE for the promotion of healthy dietary intake and physical activity behaviours in children with the intention of including context-related items providing challenging situations for the respondent. The Bohman scale yielded a four-factor solution with factors corresponding to facilitating or impeding PSE for healthy dietary intake behaviours (factors 1 and 2) and physical activity behaviours (factors 3 and 4). In comparison with the Bohman scale, the scales of this study have a lower number of items, and show a better model fit based on RMSEA, CFI, and SRMR. The Norman scale, with three factors measuring PSE to promote children’s healthy behaviours, and limit unhealthy dietary intake and PA behaviours, included a few items that included challenging situations, but the scale has not been psychometrically tested using CFA [30].

Regarding scales that have been tested for criterion validity, just a few have done so using objectively measured PA. Adkins et al. [31] measured PA in 8- to 10-year-old girls after school and found significant correlations between PSE and children’s PA. However, the scale is comprised of unspecific and non-contextualised PSE items without any challenging aspects for the parents, for example: “Can you get your daughter to go for a walk with you?”. Bohman et al. [20] and Norman et al. [30] have both used accelerometery in the evaluation of criterion validity of PSE scales. Bohman et al. found correlations between PSE and PA of *p* = 0.02, whereas Norman et al. found no significant correlations. However, both studies used child PA measured over the entire day, not just during time spent in the home environment, where parents have a much greater possibility to influence their children’s behaviour. In addition, neither of the scales adhered fully to Bandura’s guidelines for self-efficacy scale development, regarding the inclusion of context-related items providing challenging situations for the respondent.

### Usefulness of the scales for research

The brief PSE scales developed and assessed in this study may be useful for researchers studying PSE as either an outcome or a mediator, or in particular when the aim is to compare PSE between different groups. Interventions focusing on children’s weight, healthy dietary intake and physical activity behaviours or PSE often struggle with finding measurement tools that involve as little burden as possible for participants. The scales described here include only three items per behaviour, which is an adequate number of items to represent a construct, but less, and thus less burdensome, than many other PSE scales [19–21, 24, 27–31], including the Wright scales [26], even those specifically validated for use in intervention studies [20, 21, 25–28, 30].

An important aspect of any scale’s usefulness in research is that the underlying concept captured by the scale means the same thing across groups and therefore functions equally across groups, i.e. is invariant [37, 38]. We found evidence for the invariance of the scales in this study. Thus, the scales can be used to compare PSE scores between mothers and fathers, between parents with children of normal weight or obesity, and between parents with different educational levels, assuming a study has sufficient power and sample size. We found one exception, and recommend therefore that PSE scores for unhealthy behaviours across parental educational level should currently be performed with the omission of item 9 (see Table 1). However, future work should focus on further development of item 9 in order to form an invariant, brief scale for soft drinks which includes three items.

### Usefulness of the scale for clinicians

Clinicians who meet families that need support with e.g. the prevention of unhealthy weight development may find the brief scales useful for their practice. The respondent burden is low yet they can help clinicians understand the parents’ self-efficacy levels, which may help the clinician tailor their counselling. For example, if a parent scores low on a scale, this is a clue that the parent may need further support in the targeted situation. Conversely, if the parent scores highly, this is an indication that effort may be better spent on other aspects of behaviour formation and changes for the child in the home environment. Such aspects, according to Social Cognitive Theory [15], can comprise e.g. parental outcome expectations, or child observational learning which can be influenced by parental modelling of behaviours for the child.

### Strengths and limitations

A strength of this study is that the scales are tested for invariance. This provides important information about the validity of the scales and increases their usefulness for both research and clinical practice. A further strength is the inclusion of both mothers and fathers. It is common for PSE to be evaluated with mothers exclusively [19, 20, 27], or to only include a smaller proportion of fathers [24, 26, 29]. In Sweden, as in many other countries, fathers play an important role in child rearing and thus are important to include in the evaluation of scales related to parenting. Also, the scales were tested in a parental sample in a setting characterised by low socioeconomic position and with a variation of geographical backgrounds. One limitation is that only partial invariance was supported regarding parental educational level regarding measurements for children’s unhealthy behaviours. A further limitation of the study is the sample size in the invariance testing across child weight status where the group of parents of children with overweight or obesity (*n* = 60) can be considered small. Further research should focus on invariance testing across parental characteristics in larger sample sizes. Also, two error terms for items in the CFA model for unhealthy behaviour were correlated. Error terms for PSE for limiting soft drinks intake (items 7 and 9), both correlated with PSE for limiting intake of sweets (item 10). Items 7 and 10 had a very similar wording, and a further development of the scales could focus on revising these items. In addition, the mean values for items in the scale measuring PSE for limiting intake of sweets were in a somewhat narrow range between 6.80 to 8.59, and higher than the other scales of the study. This indicates that parents were generally confident that they could limit their child’s sweet intake and further development of the scale could try to make the situations in the items even more challenging, in order to capture a greater variation in PSE. The generalizability of the scales presented in this manuscript need to be considered when using it in settings other than the one targeted in this study and cross-cultural adaptations should be made necessary when the scales are to be used in cultural contexts other than the Swedish one. A further limitation to the generalizability is that, as this study was nested in the larger HSSP study, it was conducted in disadvantaged areas. However, 60% of the parents participating in the present study had attained higher education than post-secondary school (Table 2), which is comparable to the national average of 57% [59]. Further, the lack of assessment of reliability in the form of test-retest, and assessment of predictive validity of the scales are limitations to consider.

## Conclusion

This study found support for moderate to excellent construct validity for four brief scales to measure parental self-efficacy for encouraging children’s healthy dietary intake and physical activity behaviours. In addition, measurement invariance was established regarding parental sex and child weight status, and partial measurement invariance regarding parental level of education. These brief scales can be used in research where the minimal participant burden, and ability to detect differences between parental sub-groups are positive attributes. In addition, these scales can be used in clinical practice to inform clinicians where parents may need further support regarding self-efficacy in relation to children’s obesogenic behaviours.

## Supplementary Information

**Additional file 1.**

## Data Availability

The datasets generated and/or analysed during the current study are not publicly available because of ethical reasons, where public availability would compromise participant privacy, but are available from the corresponding author on reasonable request.

## Abbreviations

PSE: Parental self-efficacy
PA: Physical activity
HSSP: Healthy School Start Plus
MVPA: Moderate to vigorous intensity
CFA: Confirmatory factor analysis
CFI: Comparative fit index
RMSEA: Root mean squared error of approximation
SRMR: Standardised root mean residuals
