# Supplementary material for: Brief parental self-efficacy scales for promoting healthy eating and physical activity in children: a validation study
Source: BMC Public Health. 2021 Mar 19;21:540. doi: 10.1186/s12889-021-10581-7 (PMC7980573; doi:10.1186/s12889-021-10581-7)
Supplement: Supplementary file 1 — Additional file 1. [file 12889_2021_10581_MOESM1_ESM.docx]

Supplementary file 1

| Description of questionnaire | |
| --- | --- |
| Behaviour/item  Basic stem: *“How certain are you that you can…”* |  |
| Healthy behaviours |  |
| Physical activity |  |
| *“…. make sure that your child is physically active in such a way that he/she gets a little sweaty or out of breath for at least 1 hour during the day?”* |  |
| 1. …when there are many other things to do |  |
| 2. …when you are tired |  |
| 3. …when the weather is bad |  |
| Vegetables |  |
| *“…influence your child to eat at least 2 servings of vegetables at home each day?”* |  |
| 4. …when you are too tired to prepare them |  |
| 5. …when other family members don’t want to eat vegetables |  |
| 6. …when you are eating out at a restaurant |  |
| Unhealthy behaviours |  |
| Soft drinks |  |
| *“…limit how much soft drinks and sap your child drinks so that your child does not drink more than 2 glasses (3 decilitres) per week”* |  |
| 7. …when other family members drink it |  |
| 8. …when you eat at a restaurant |  |
| 9. …when your child wants it |  |
| Sweets |  |
| *“…limit how much sweets/chocolate your child eats so that your child does not eat more than 100 grams/1.5 decilitre sweets per week”* |  |
| 10. …when other family members eat it |  |
| 11. …when your child refuses to eat food |  |
| 12. …when your child wants it |  |
|  |  |
